# Coarse Electrocorticographic Decoding of Ipsilateral Reach in Patients with Brain Lesions

**DOI:** 10.1371/journal.pone.0115236

**Published:** 2014-12-29

**Authors:** Guy Hotson, Matthew S. Fifer, Soumyadipta Acharya, Heather L. Benz, William S. Anderson, Nitish V. Thakor, Nathan E. Crone

**Affiliations:** 1 Department of Electrical and Computer Engineering, Johns Hopkins University, Baltimore, Maryland 21218, United States of America; 2 Department of Biomedical Engineering, Johns Hopkins University, Baltimore, Maryland 21205, United States of America; 3 Department of Neurosurgery, Johns Hopkins University, Baltimore, Maryland 21287, United States of America; 4 Department of Neurology, Johns Hopkins University, Baltimore, Maryland 21287, United States of America; University of Texas Health Science Center at San Antonio, Research Imaging Institute, United States of America

## Abstract

In patients with unilateral upper limb paralysis from strokes and other brain lesions, strategies for functional recovery may eventually include brain-machine interfaces (BMIs) using control signals from residual sensorimotor systems in the damaged hemisphere. When voluntary movements of the contralateral limb are not possible due to brain pathology, initial training of such a BMI may require use of the unaffected ipsilateral limb. We conducted an offline investigation of the feasibility of decoding ipsilateral upper limb movements from electrocorticographic (ECoG) recordings in three patients with different lesions of sensorimotor systems associated with upper limb control. We found that the first principal component (PC) of unconstrained, naturalistic reaching movements of the upper limb could be decoded from ipsilateral ECoG using a linear model. ECoG signal features yielding the best decoding accuracy were different across subjects. Performance saturated with very few input features. Decoding performances of 0.77, 0.73, and 0.66 (median Pearson's *r* between the predicted and actual first PC of movement using nine signal features) were achieved in the three subjects. The performance achieved here with small numbers of electrodes and computationally simple decoding algorithms suggests that it may be possible to control a BMI using ECoG recorded from damaged sensorimotor brain systems.

## Introduction

The brain-machine interface (BMI) is a tool to replace areas of human functionality lost due to trauma or degenerative disease. To date, BMIs have been used with humans to control cursors [1]–[3], move robotic limbs [4]–[7], and allow patients with locked-in syndrome to communicate with the outside world [8]. Electrocorticography (ECoG) electrodes are implantable, non-penetrating, and occupy a unique space between the high fidelity of cortex-penetrating microelectrode arrays recordings and the low fidelity and bandwidth of noninvasive EEG. A long history of work with EEG and ECoG signals has highlighted mu (8–12 Hz), beta (14–30 Hz) [9], [10] and high gamma (>70 Hz) [11] band signals as indices of cortical motor processing. More recent work has shown that ECoG signals may also contain movement-related information in their smoothed amplitudes, or local motor potentials (LMPs) [12]–[14].

In most efforts to develop a brain-machine interface (BMI) to restore upper limb function, the design has assumed a normally functioning brain as the source of neural control signals, with the BMI serving to bypass lesions of neural pathways connecting the brain to its muscle effectors. However, a large patient population with hemiplegia from strokes and other cerebral lesions may also benefit from BMIs [15]. In these patients, a variety of approaches are already being used or developed to restore function. These include intensive neurorehabilitation [16]–[18], direct current stimulation of the motor cortex [19], [20], and neurobiological therapies, e.g. infusions of stem cells and growth factors, aimed at promoting neurogenesis in the damaged hemisphere [21]–[23]. It might also be feasible to restore function via neural signals from the undamaged hemisphere [24]–[26]. However, cerebral representations for upper limb control are primarily contralateral rather than ipsilateral, and the risks associated with surgical implantation of electrodes over healthy, functional cortex may not outweigh the potential benefits. To overcome these limitations, an alternative approach could be to utilize residual sensorimotor systems in the damaged hemisphere. This approach would improve the risk-benefit ratio because subdural electrodes for an ECoG-based BMI could be implanted along with other invasive experimental therapies. Furthermore, training of the BMI, along with intensive neurorehabilitation, could generate neural activity encouraging neurogenesis and plasticity in residual brain tissue, potentially working synergistically towards functional recovery. However, in patients with total paralysis of the contralateral upper limb, arguably the population to whom a BMI would be most attractive, it would be challenging to train the BMI's decoding algorithms. To overcome this limitation, subjects could be trained by imagining their own arm moving [27], viewing arm movements [28], [29], being passively moved [30], or even performing movements with the ipsilateral arm [31]. The neurophysiological correlates of reaching movements in residual motor systems of the ipsilateral cortex therefore warrant investigation.

The descending corticospinal tract is not solely comprised of axons that cross the midline. A minority of the fibers also arise from ipsilateral cortex [32]. Ipsilateral wrist movements may be impaired in hemiparetic subjects with unilateral brain lesions [33]. A substantial portion of PMd and a nontrivial number of M1 cells display tuning to ipsilateral arm movements in primates [34]. Indeed, the efficacy of rehabilitative therapies in many patients with upper limb paralysis from unilateral brain lesions [18] implies not only that functional reserve and potential for plasticity is present in the lesioned hemisphere, but also that ipsilateral descending motor pathways can be utilized for upper limb control. The degree to which arm movements can be decoded from the neural activity of a damaged ipsilateral cortex, however, is not well known and is of particular interest for training an upper limb BMI in patients with total upper limb paralysis from a stroke or other unilateral cerebral lesion.

Reach trajectories have been decoded extensively in nonhuman primates performing center-out reaches with microelectrode arrays [35]–[38], and more recently in humans and primates implanted with ECoG grids [39]–[42], using neural signals contralateral to the reaching arm. Previous work has also shown the potential for extracting neural correlates of overt and imagined hand movements from intact ipsilateral cortex in humans using ECoG [31], [43] and EEG [44]. However, it has not previously been shown if neural signals from damaged cortical areas can retain any information about ipsilateral arm movements. In this study, we investigated the neural correlates of ipsilateral reaching movements in human subjects with lesions in the hemisphere recorded with ECoG. In two subjects, these lesions disrupted motor pathways and were associated with motor impairment of the contralateral arm. In one subject, the lesion involved superior parietal lobule, likely including the human homolog of parietal reach region, but there was no associated motor impairment.

## Materials and Methods

### Data Acquisition

Three subjects, two male and one female aged 24–36 years, were implanted with ECoG arrays prior to surgical resection of their seizure foci. Additional subject information can be found in the supporting materials. Grid placement was determined solely on the basis of their clinical need without any influence from this study. Only subjects with motor cortex coverage were considered for this study. Table 1 summarizes the clinical and demographic information associated with each study participant. All subjects gave written informed consent and the study was conducted under a protocol approved by the Johns Hopkins Institutional Review Board (IRB). ECoG grids consisted of rectangular arrays of platinum electrodes (Adtech Medical Instrument Corp; Racine, WI) with 2.3 mm diameter and center-to-center spacing of 10 mm. Electrodes were embedded in a Silastic sheet and implanted on the brain in the subdural space. Arrays of nonpenetrating micro-ECoG leads (75-micron diameter, 0.66-mm spacing) were also implanted in each of the three subjects, but in subjects 1 and 2, these micro-ECoG leads were not over motor areas and therefore not included in our analysis. Of the implanted electrodes, 44–48 per subject were analyzed for this study. A 4×4 array of 16 micro-ECoG leads was included in the analysis for subject 3. These micro-ECoG leads were located in posterior middle frontal gyrus, anterior to electrodes where electrocortical stimulation mapping (ESM) affected motor function, and their signals did not produce notable decoding results. The tests described below were part of a larger battery of research testing. These tests were conducted between two and seven days after implantation, and removal of the grids occurred at the time of seizure focus resection, seven to eight days after implantation.

**Table 1:** Subject Demographic and Clinical Information.

Subject	Age (years)	Handedness	Gender	ECoG coverage	Seizure focus/pathology	Neuro deficit	Reaching arm
1	36	Right	Male	Right frontal-parietal	Right frontal oligodendroglioma	Left hemiparesis	Right
2	24	Left	Male	Right parietal-occipital	Right occipital	Left inferior quadrant visual defect	Right
3	30	Left	Female	Left frontal-parietal	Stroke involving posterior limb of left internal capsule	Right spastic hemiplegia	Left

**Figure 1:**
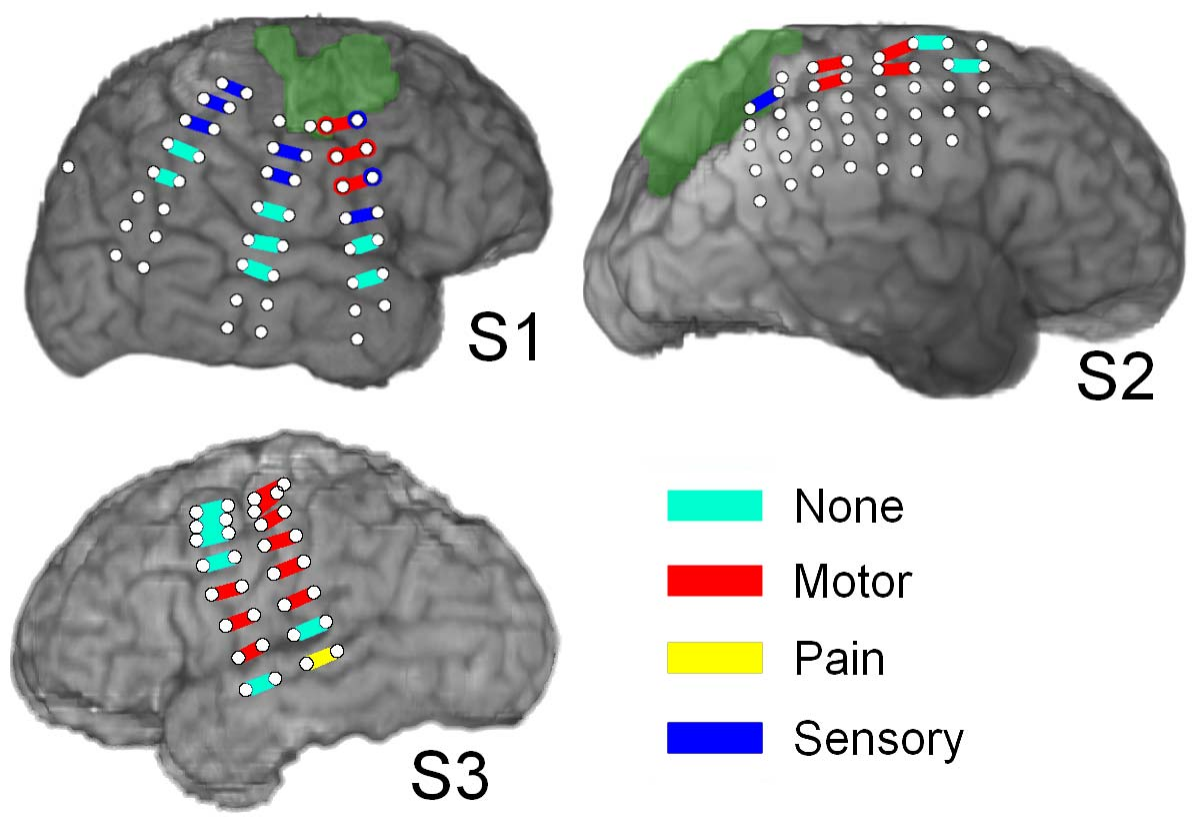
Reconstructions of electrode placements with ESM results. The grids shown are the subset of implanted electrodes that were recorded from during this study. The green highlighted areas correspond to regions of cortical lesions. The lesion in subject 3 could not be seen on the brain surface rendering because it was located beneath the surface of the brain. Colored rectangles joining electrodes imply that bipolar stimulation was applied to that pair of electrodes. In subject 1, several electrode pairs were further investigated by performing unipolar stimulation relative to a distant reference electrode. The results of this unipolar stimulation are shown with colored circles surrounding the electrodes.

**Figure 2:**
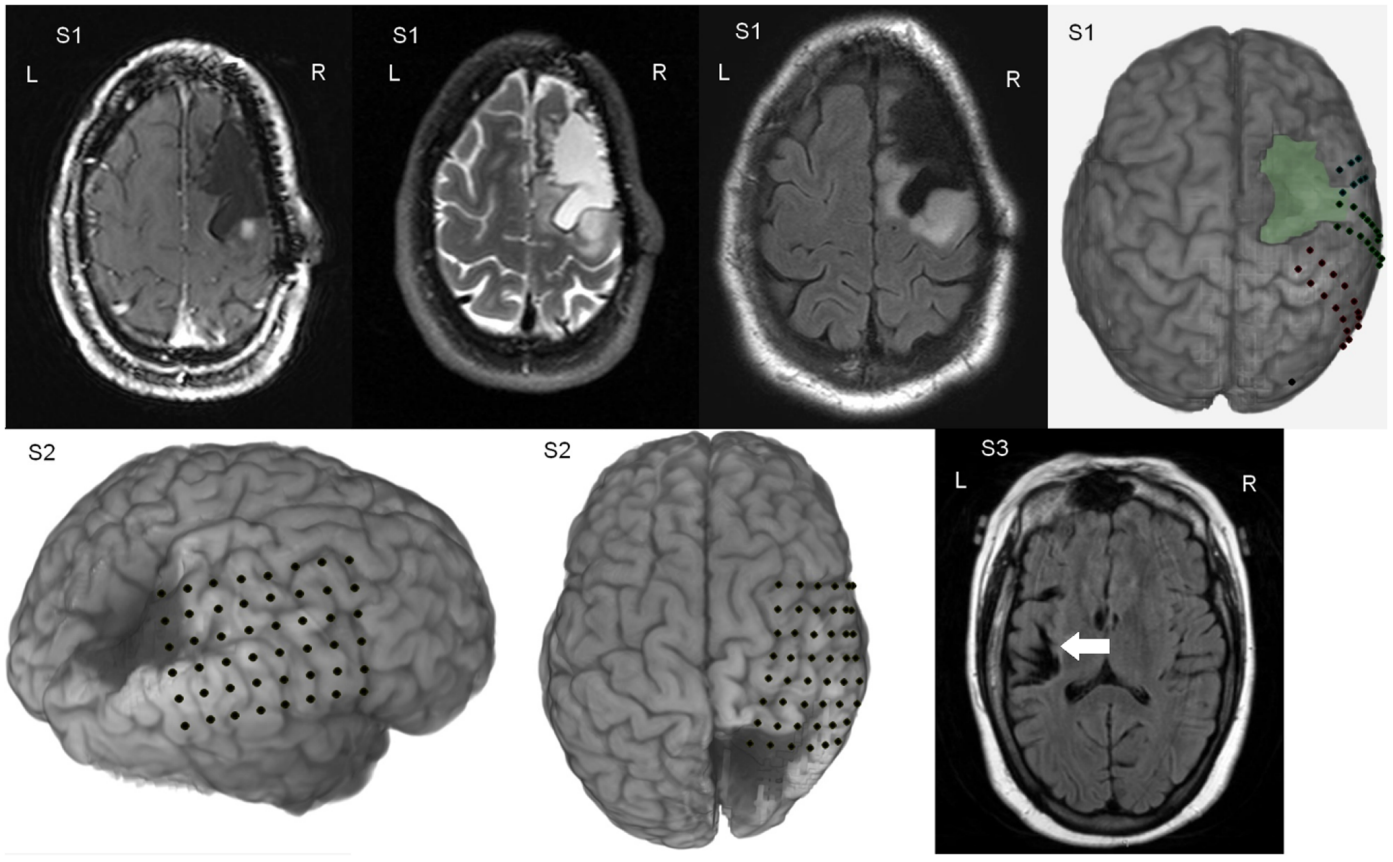
Presurgical MRI and brain reconstructions. Reconstructions are shown for subject 1 (first row) subject 2 (bottom left and middle) and subject 3 (bottom right). The previous resection margins anterior to the precentral gyrus in subject 1 are highlighted in green in the upper right. Superior oblique and top axial views of the reconstruction for subject 2 show the lesion from different viewpoints. Pre-surgical MRI (FLAIR) of subject 3 reveals a lesion of posterior left insula also involving left internal capsule.

ECoG signals were referenced to an inactive intracranial electrode and recorded by a 64-channel Neuroscan Synamps^2^ (Compumedics; Charlotte, NC) for subject 1 and 2; ECoG signals from subject 3 were also referenced to an inactive intracranial electrode, but were recorded by a 128-channel NeuroPort system (BlackRock Microsystems; Salt Lake City, UT). Signals were digitized by the Neuroscan system at 1 kHz after being band-pass filtered between 0.15 and 200 Hz (subject 1 and 2). Signals recorded by the BlackRock system were band-pass filtered in analog with a low-pass cutoff of 7,500 Hz and a high-pass cutoff of 0.3 Hz, digitized at 30 kHz, then downsampled to 1 kHz. Anatomical reconstructions of each subject's electrode placement were generated by volumetrically co-registering post-implantation brain CT with high-resolution pre-implantation brain MRI images using BioImage (Yale, [45]). Additional information from surgical photos, post-implantation skull X-rays in the antero-posterior and lateral axes, and ESM were used to verify electrode locations. Reconstructions of electrode locations, overlaid with the color-coded results of ESM, are shown in Fig. 1. Fig. 2 displays the locations of the cortical lesions for each subject.

The three-dimensional positions of the subject's shoulder and arm were tracked optically using the Northern Digital Optotrak system with a sampling frequency of 100 Hz. Neural data and movement data were synchronized using parallel port triggers sent simultaneously from the experimental computer over a split cable to the Neuroscan (subject 1 and 2) or NeuroPort (subject 3) amplifier's external trigger inputs and the Optotrak Data Acquisition Unit (ODAU).

### Experimental Paradigm

**Figure 3:**
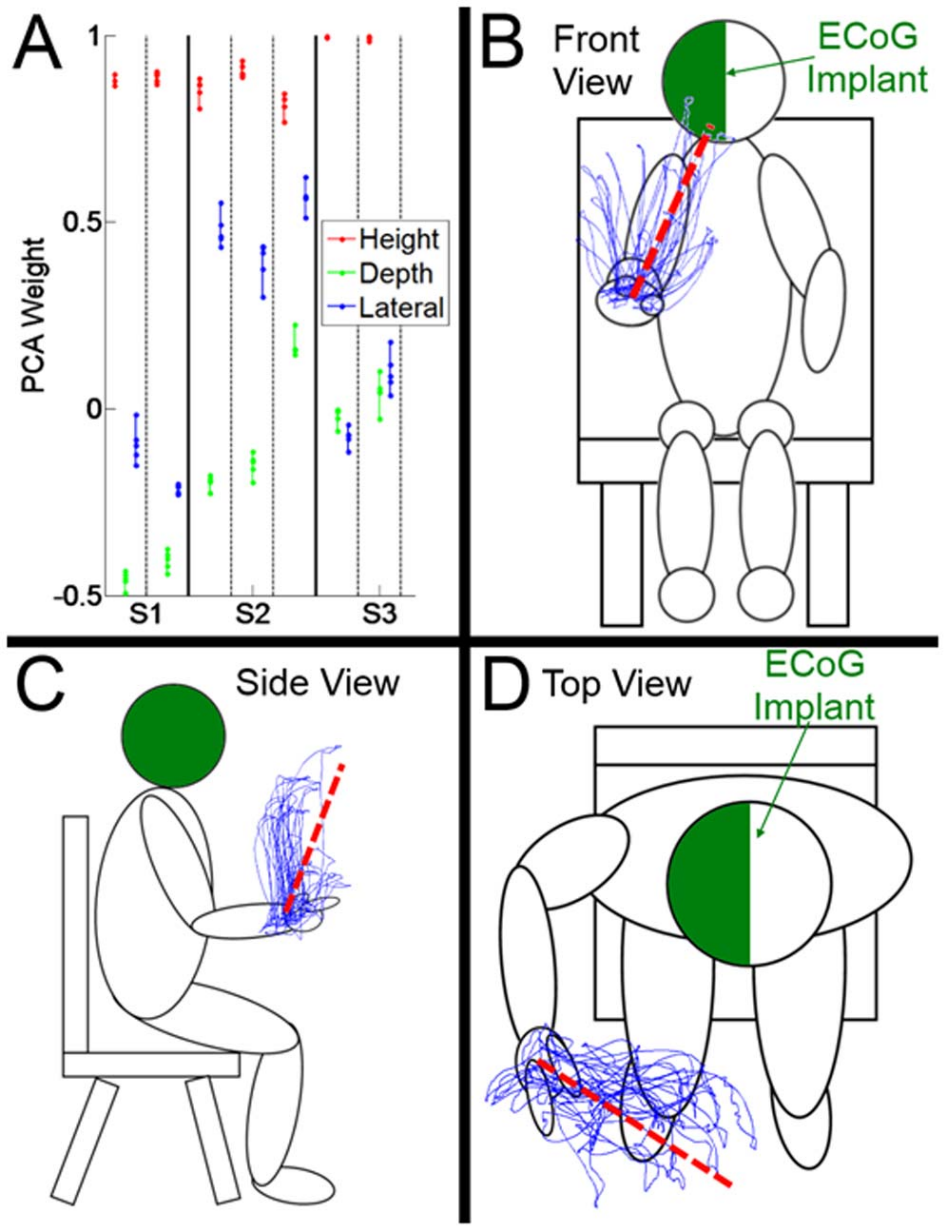
Subject Kinematics and Experimental Paradigm. (A) Shows the weights given to each of the three dimensions (height, depth, and lateral) when computing the first PC of the movement kinematics. Each point represents the PC weight calculated using a different cross-validation. (B–D) Show different views of the experimental paradigm and example kinematics from one of the subjects' sessions. The red dashed line represents the first PC plotted in Cartesian space for that same session.

Subjects were instructed to make reaches to the tip of a long wooden dowel being moved by the experimenter in three-dimensional space. Subjects used the arm ipsilateral to the ECoG electrode implantation. Each reach was either terminated by touching the dowel with the subject's pointing index finger (subject 1, session 1; subject 2; subject 3) or with an index-thumb pinch (subject 1, session 2). The subject returned his or her hand to a comfortable resting position following completion of each reach to the target. The subject rested his or her hand in the home position for a variable amount of time (0–16.7 seconds), after which the target was moved to another point in three-dimensional space. The position of the target in three-dimensional space was determined by the experimenter in an attempt to probe the natural workspace of the subject's upper limb as thoroughly as possible. The target tended to be placed in front of and above the subject's rest position, but varied in the lateral (i.e., left or right) directions. The durations of the reaches performed by subjects 1, 2, and 3 were 3.2–6.2, 2.0–5.0, and 1.2–5.3 seconds, respectively, with median durations of 4.7, 3.0, and 2.7 seconds. The volume encompassed by the subject's workspace is detailed in S1 Table. A visual depiction of the workspace and experimental setup is shown in Fig. 3, along with example subject kinematics. The subject was cued to begin each reach once the target had stopped moving to its new location. Our analysis included two blocks of trials each from subject 1 and 3 and three blocks of trials from subject 2; details are listed in S2 Table. The cuing dowel was tracked using the same Optotrak system that was used simultaneously to track the subject's shoulder and hand, with a sampling frequency of 100 Hz. Because the tracked target tip was occluded during manipulation by the subject, the position of the distal tip was estimated using proximal sensor locations and the known, fixed distance between the sensors and the end of the dowel.

### Neural Data Feature Extraction

Recorded ECoG signals were imported into MATLAB (MathWorks, Natick, MA) and analyzed using the Signal Analysis Toolbox. These signals were re-referenced to a common average reference (CAR) to avoid spatial biases from varying distances between active and reference electrodes [46]. The CAR-filtered signals were then filtered forward and backward (to avoid phase distortions) using a Hamming window and a series of 400-order FIR filters with bandpass cutoff frequencies corresponding to delta (0–4 Hz), theta (4–8 Hz), mu (7–13 Hz), beta (14–30 Hz), low gamma (30–50 Hz), high gamma 1 (70–110 Hz) and high gamma 2 (130–200 Hz) bands. The frequency limits for these bands were chosen to ensure inclusion of all relevant frequencies and to avoid 60 Hz line noise and its harmonics. The filtered signals were then down-sampled to 100 Hz to match the sampling frequency of the kinematic data. These signals were squared to yield power in each band, then passed through a smoothing integrator (1 second moving average, applied forward and backward), and log-transformed to approximate normal distributions. Transients in the neural data induced by the filtering operations at the beginning and end of each block of trials were not analyzed in this study.

In addition to frequency domain features, previous work indicated that smoothed time domain features contained information related to movement [12], [14]. These features, known as local motor potentials (LMPs) were extracted in this study using a moving average filter of two seconds, applied forward and backward to remove phase distortions. All feature extraction was done in a non-causal manner (using data from both the past and the future) to avoid introducing a group delay as a confound in our results.

### Decoding Model Construction and Evaluation

The kinematics were modeled as a linear combination of the input features with a constant offset and a Gaussian noise term: 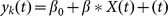, where 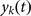 denotes the predicted output of the *k*th dimension of the kinematic output, 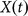 is the vector of feature values at time 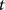, 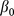 is the constant offset term, and 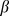 is the vector of linear weights applied to the features, and 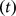 is zero-mean Gaussian noise. The parameters of this model were estimated using the glmfit function in MATLAB.

All models were tested under fivefold cross-validation. Pearson's correlation (*r*) between observed and predicted kinematics was used to quantify model accuracy. Feature selection and model training were performed on 80% of each block of trials, and the remaining 20% was used only for evaluation. Each training session contained at least 18 movements, and each testing session contained at least 4 movements. Chance decoding performance was calculated for each session by randomly shuffling the extracted and smoothed neural features 1024 times, similar to the random shuffle procedure done in [47]. This shuffled data was then used for testing and training with fivefold cross-validation. The distributions of the decoding accuracies with actual and shuffled data were compared with a Bonferroni-corrected Wilcoxon test.

Decoding accuracies were transformed into z-scores using the Fisher transformation, 
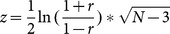
, where 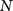 is the number of points used to calculate the correlation. These transformed accuracies were used as inputs to a two-way ANOVA with the number of inputs and feature type (e.g., theta features only, all features) as independent factors. A post-hoc *t* test was done using the Dunn-Sidak correction [48] for multiple comparisons to determine which numbers of inputs and/or which feature types significantly differed, if any.

Both the neural features (after all preprocessing) and the kinematic data were normalized by subtracting the means and dividing by the standard deviations. The means and standard deviations were recomputed during every fold of cross-validation using only the training data. This normalization was done to ensure the magnitude of the features did not impact how heavily they were weighted in the decoding models.

### Principal Component Analysis

While Cartesian coordinates for arm position (i.e., vertical, lateral, and depth) are orthogonal, constraints on the positioning and capabilities of the subjects resulted in an inability to fully probe these axes of the workspace independently. The kinematics of the resulting movements in this space were correlated to the extent that presenting decoding results in Cartesian axes would be somewhat misleading. We therefore used principal component analysis (PCA) to decorrelate the kinematic dimensions by projecting the 3D arm position onto another set of orthogonal axes. These decorrelated kinematics, or principal components (PCs), accounted for decreasing proportions of the variance in the original signals. The first PC was therefore oriented in the direction of largest variance (i.e. the vector along which the subject moved the most), while the second PC was oriented in the direction of the most *remaining* variance, etc.

The coefficients used for the PCA transform were recalculated for each fold of cross-validation using only the normalized training data. In all three subjects, decoding accuracy of the first PC was greater than the second and third PCs (*p*<0.05, ANOVA with Dunn-Sidak post-hoc). All but one of the models built on the second and third PCs across all subjects were indistinguishable from chance. We therefore focus on the decoding accuracies of the first PC in this study.

### Model Input Feature Selection

Models with increasing numbers of input features were trained; inputs were added in decreasing order of their correlation with the kinematic variables in the training set. Correlations were calculated for the kinematic signal lagged with respect to the neural features at time lags varying between ±1 second at 50 ms intervals. A single spectral domain or time domain signal was only included once at its best lag. Results from model orders of up to 40 are reported below. Additionally, restricted models were trained from single feature types (e.g., high gamma 1 features only). Performance of these restricted models is also reported below in comparison to the full model performance for model orders one of up to 40. An analysis of the performance saturation of these models with increasing numbers of inputs was performed with a Kruskal-Wallis non-parametric one-way analysis of variance. The least significant difference post-hoc test was used to determine the first number of inputs at which additional inputs did not statistically increase performance.

The large smoothing window applied to the extracted features meant it was possible that changes in the neural features could occur substantially before or after their associated movements but still yield a high correlation with the movements. To investigate whether the feature changes preceded or followed the associated movements, we calculated correlations every 50 ms between the extracted features and the first principal component of movement offset by at most 1000 ms relative to one another.

## Results

### Feature Selection

**Figure 4:**
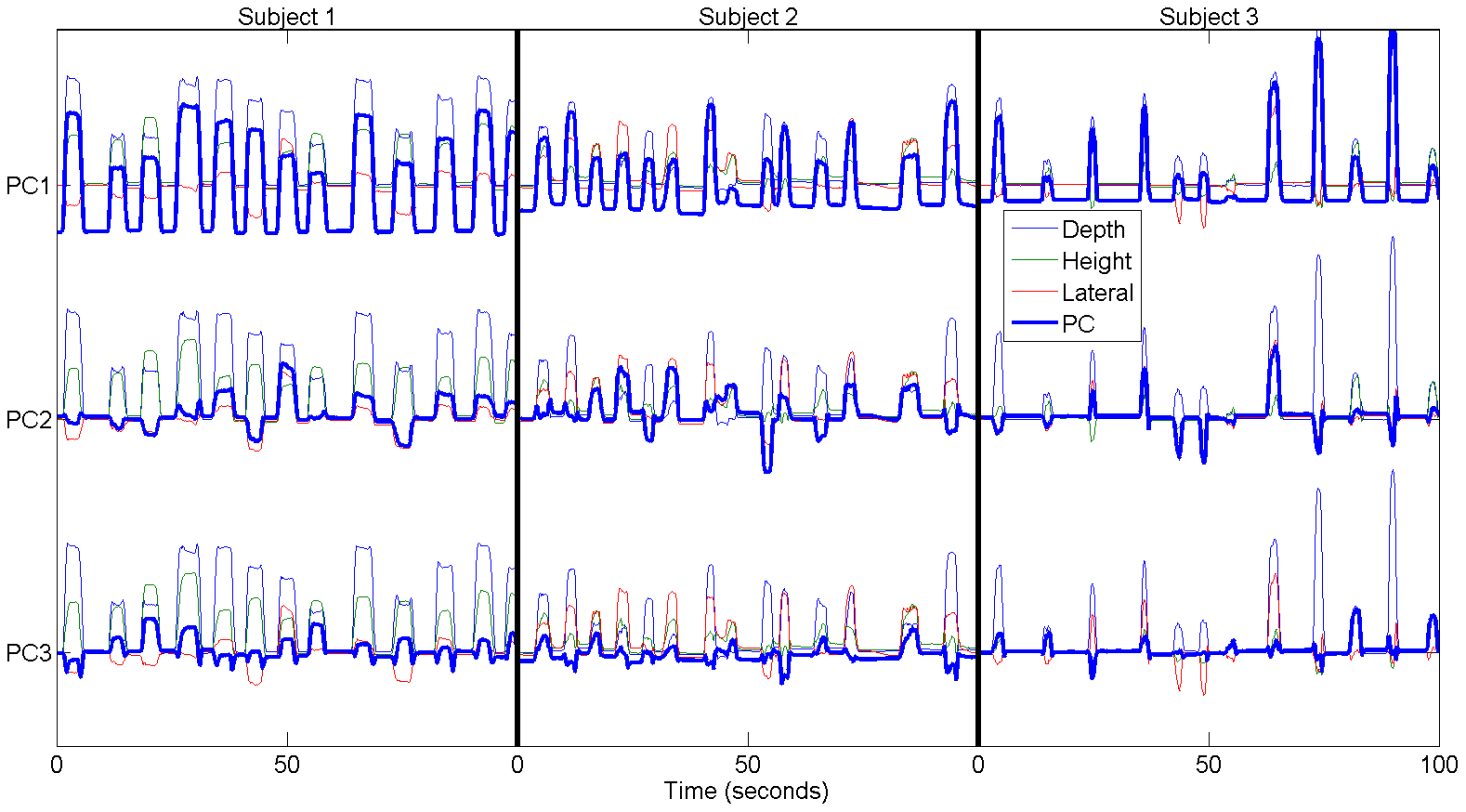
Example Principal Components of Kinematics. A representative example of the first, second, and third principal components of the kinematics from each subject, overlaid with the original kinematics.

**Figure 5:**
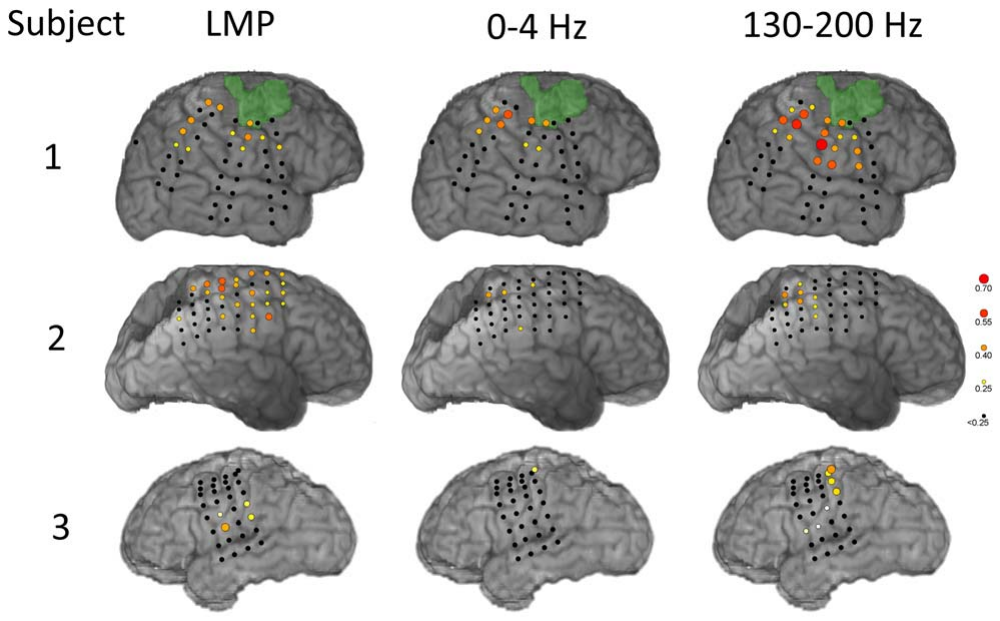
Anatomical patterns of average correlations between representative ECoG signal features and the first PC of the hand position. Correlations were calculated at each feature's best lag (within ±1 second) relative to the movement data. Color-coded circles corresponding to maximum correlations, averaged across experimental sessions, are shown for each subject.

The extracted signal features had substantial correlations with the first PC of the ipsilateral hand position, and the maximally correlated feature types varied across subjects. The three-dimensional kinematics and their contributions to the first PC are shown in Fig. 3. Examples of how each of the PCs relate to the original kinematics is shown in Fig. 4. Fig. 5 illustrates the correlations between different ECoG signal features (LMP and bandpass power) and the first PC of hand position in each subject. The magnitudes of these correlations are color-mapped onto the ECoG recording sites from which the features were derived. The lagged correlations were calculated at 50 ms intervals between −1 and +1 seconds relative to the hand kinematics. The peak correlations (i.e., across all lags) were averaged across sessions for each electrode. Subject 3 contained micro-ECoG electrodes, but these are not depicted due to their lack of correlation.

**Figure 6:**
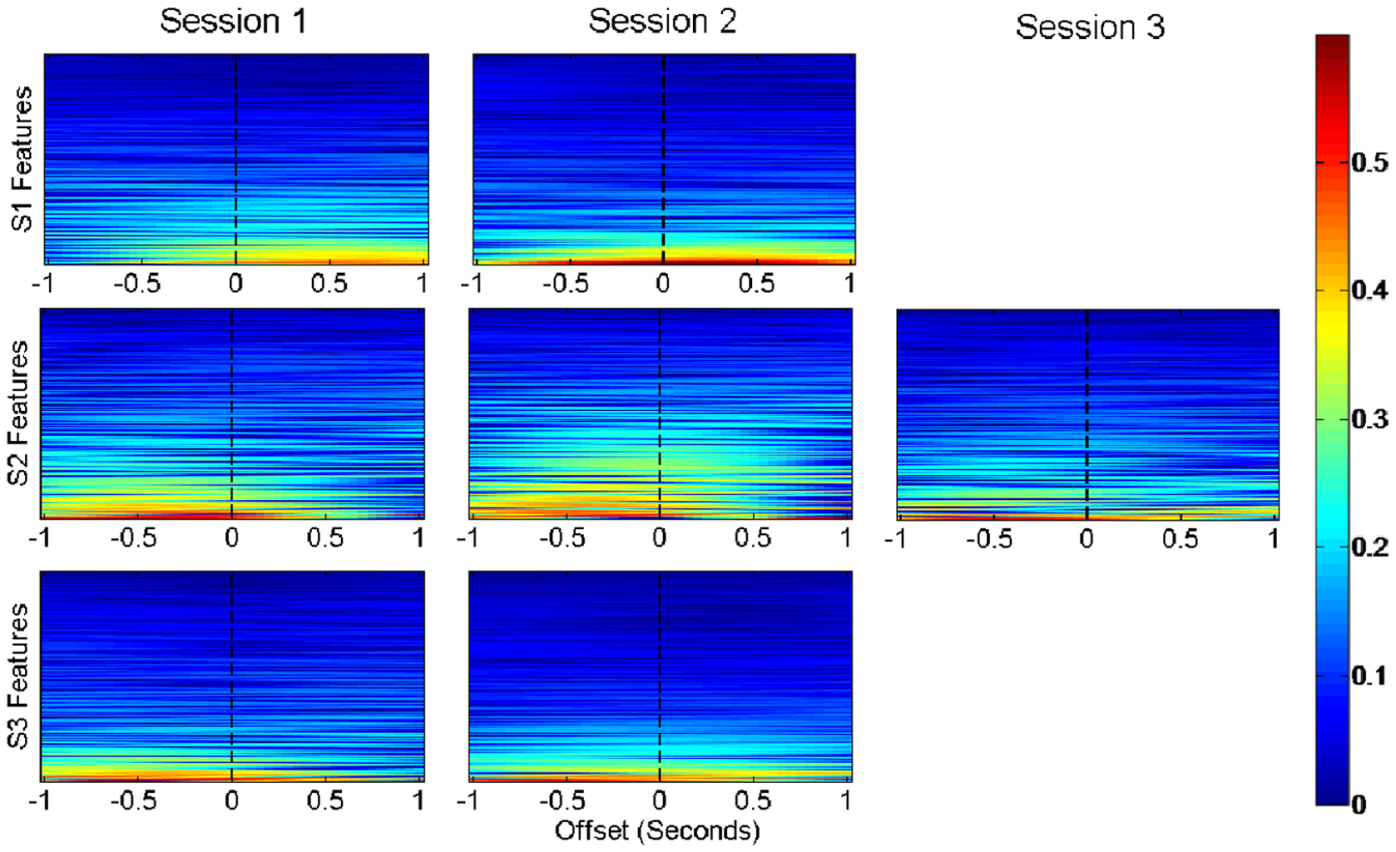
Correlations between kinematics and neural features at different time lags relative to one another. Each row corresponds to one of eight extracted features from one electrode, resulting in 384, 352, and 360 rows for subjects 1, 2, and 3 respectively. The x-axis corresponds to the lag between the neural feature and the reach position. A negative lag represents changes in the neural features occurring before the corresponding kinematics, and a positive lag represents feature changes after the kinematics. Rows are ordered by the magnitude of their peak correlation. Correlations between the hand kinematics and the features were calculated every 50 ms between leads or lags of one second.

The large smoothing window applied to the extracted features meant it was possible that changes in the neural features could occur substantially before or after their associated movements but still yield a high correlation with the movements. To investigate whether the feature changes preceded or followed the associated movements, we plotted the magnitudes of the correlations between the neural features and the hand kinematics at different time offsets relative to one another (Fig. 6). Subject 1 typically had maximum correlations corresponding to neural features changing after the hand kinematic changes, particularly in session 1. This may indicate the activations were primarily in response to sensory feedback. Subject 2 and 3 had peak correlations when the changes in the features occurred before the kinematics.

### Multiple Inputs

**Figure 7:**
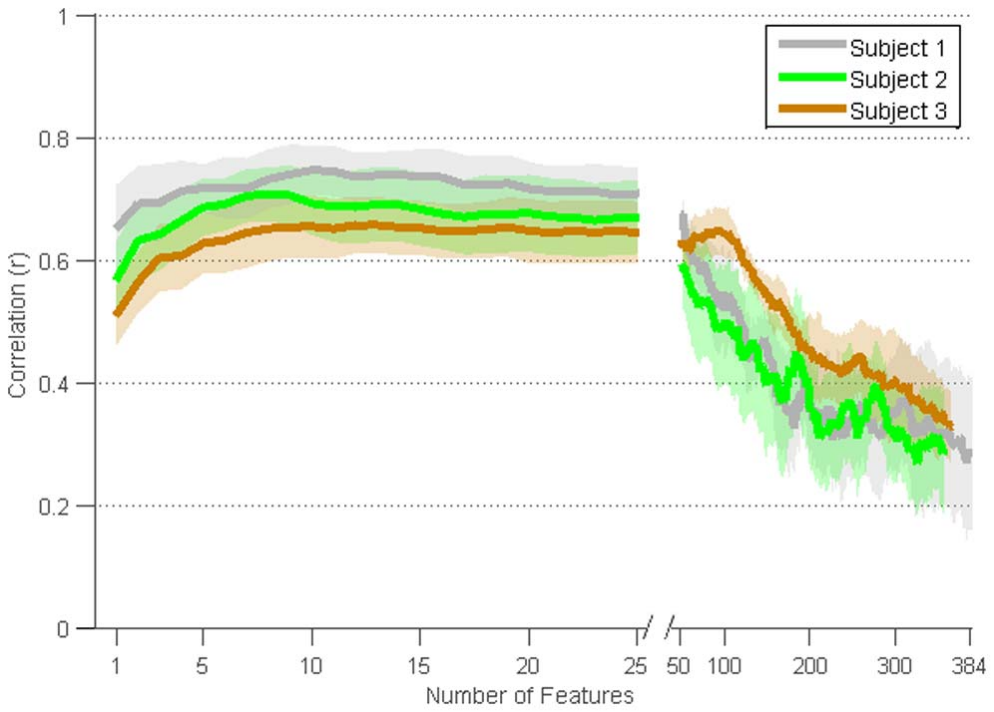
Relationship between number of model inputs (i.e., ECoG signal features) and model performance. Mean decoding accuracies of the first PC of movement across cross-validations and sessions are displayed for features chosen in decreasing order of correlation. Shading corresponds to the 95^th^ percentile confidence interval for the mean.

Fig. 7 displays the models' mean correlations with the first PC of movement as a function of the number of model inputs. Input features were selected in order of decreasing correlation with the first PC of movement in the training set. Fivefold cross-validation was performed for each session, resulting in 10 folds for subject 1 and 3 and 15 for subject 2. For subject 1 and 3, the addition of more inputs to the model did not significantly improve model performance. In subject 2, models using a single input performed significantly worse (*p*<0.05, ANOVA with Dunn-Sidak post-hoc) than models with the best performing number of inputs.

**Figure 8:**
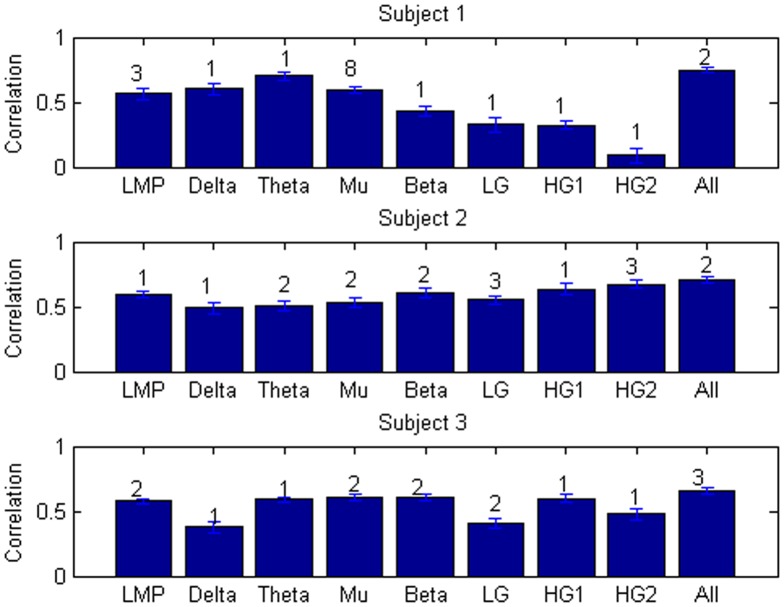
Peak decoding accuracy for each ECoG signal feature type. Between 1 and 40 recording sites were selected for each signal feature type, and the means were calculated across cross-validations and sessions. The peak accuracies are displayed here with error bars corresponding to the standard error of the mean. Numbers over each bar indicate the minimal number of features required for statistical saturation.

The input features in Fig. 7 were selected from all signal feature types. This analysis was repeated with inputs from different ECoG recording sites restricted to a single signal feature type (e.g. LMP, delta band power, etc.). Fig. 8 shows the decoding performance for models restricted to one signal feature type, using the number of inputs that saturated performance. Most of these restricted models saturated with only a single electrode, and all but one saturated with three or fewer electrodes. Decoding performance for each signal feature type was fairly unique to each subject. In all subjects, models trained with all features significantly outperformed all single feature models (*p*<0.05). Smaller ranges of time lags (e.g. −100 to 0, −200 to 0, −100 to +100, −200 to +200, and 0 to +200 ms) resulted only in small reductions in performance. The low frequency features (i.e., delta, theta, mu, and beta) and LMP performed significantly better than the high frequency features (low gamma through high gamma 2) for subject 1 (*p*<0.05). In subject 2, there was no significant difference between models trained with high gamma 1 or high gamma 2, and these sets of models outperformed all other single signal feature models (*p*<0.05). In subject 3, models trained with delta, low gamma, and high gamma 2 performed statistically worse than all other single signal feature models, which had no significant difference between each other (*p*<0.05). All *p*-values reported between feature types were obtained as a part of the two-way ANOVA with Dunn-Sidak post-hoc test described in the Methods.

**Figure 9:**
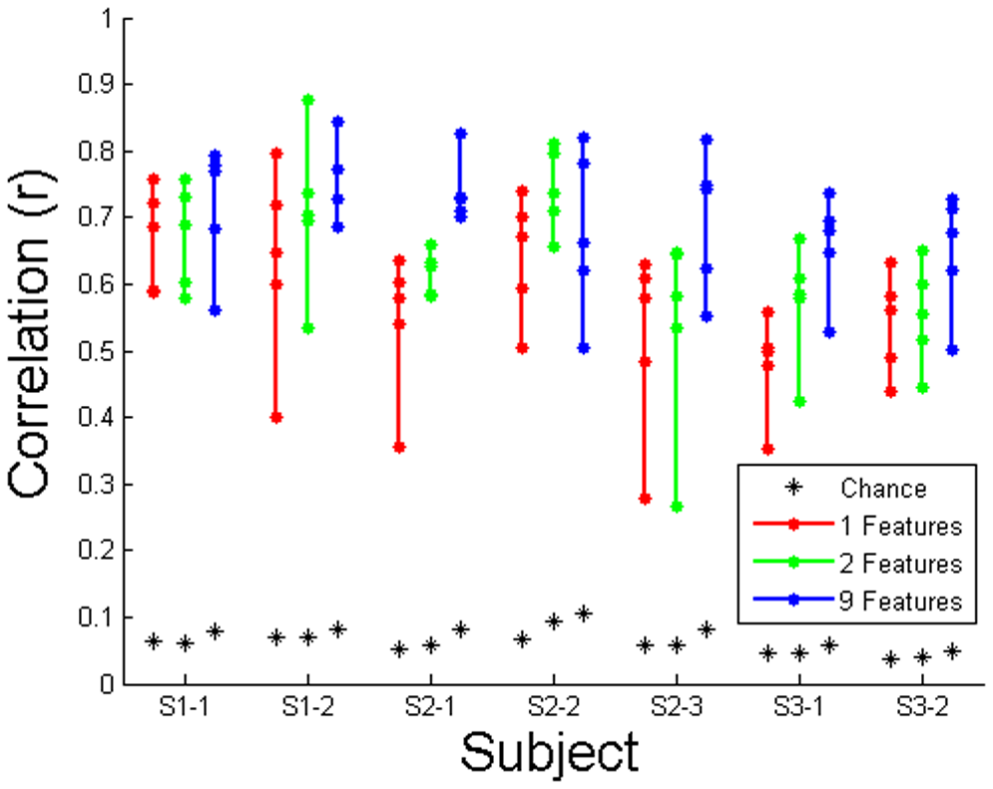
Decoding model performance across sessions for the first PC of movement. Distributions are displayed for the five cross-validations of the performance for one, two, and nine input features. S1–1 stands for subject 1 session 1, S1–2 stands for subject 1 session 2, etc. The maximum of the 1024 chance decoding attempts for all five folds with shuffled neural data is shown with an asterisk.

The decoding performance of the first PC of movement for each session is shown in Fig. 9. Results are shown for the two sessions for subject 1 and 3 and the three sessions for subject 2. Five folds of cross-validation using one, two, and nine input features are plotted for each session. During post-hoc analysis, we found that the decoding accuracy did not significantly improve (*p*<0.05, one-way ANOVA with least significant difference correction) if more signal features and/or recording sites were added to the one best model input for subject 1 or 3, or to the two best model inputs for subject 2. Models trained with nine inputs were chosen *a posteriori* as a proxy for the peak decoding accuracy across sessions and subjects, as it gave consistently high results. Decoding accuracy was found to be significantly higher than chance (*p*<0.05, Bonferroni-corrected Wilcoxon). Table 2 displays results for both raw kinematics and all PC's.

**Table 2:** Correlation between actual and decoded kinematics with and without PCA.

	Median Correlation (r)					
Subject, Session	Depth	Height	Lateral	PC 1	PC 2	PC 3
S1, 1	**0.75**	**0.71**	−0.1	**0. 73**	−0.14	0.15
S1, 2	**0.75**	**0.80**	**0.18**	**0.75**	−0.01	0.06
S2, 1	**0.65**	**0.61**	**0.64**	**0.73**	0.11	0.12
S2, 2	**0.64**	**0.41**	**0.42**	**0.66**	−0.04	0.02
S2, 3	**0.73**	0.12	0.14	**0.74**	**0.23**	−0.08
S3, 1	**0.49**	−0.05	**0.31**	**0.49**	−0.01	0.17
S3, 2	**0.69**	0.03	0.14	**0.68**	0.25	0.10

Same methods were employed as with the first PC. The 9 best correlated neural features were selected for decoding in each dimension. The median of five-folds of correlation is displayed for each session. Bold denotes a statistically significant difference from chance results (*p*<0.05, Bonferroni-corrected Wilcoxon).

S1, S2, and S3 Videos show a virtual depiction of the actual and decoded kinematics for representative folds of cross-validation for each subject. The actual and decoded kinematics were scaled and translated by the same amount to fit in the virtual workspace. The actual and decoded positions were then sent to a virtual modular prosthetic limb through VulcanX limb control software, as described in an online setting in [7], [49]. The videos do not account for any delays which would occur in an online control scenario because of the non-causal methods we employed.

## Discussion

Our findings in three different subjects show that it is possible to decode a low-dimensional representation of natural reaches from ipsilateral ECoG electrodes in the presence of damaged cortical motor systems. This decoding attained a median Pearson's correlation (*r*) between actual and predicted reach kinematics of 0.77, 0.73, and 0.66 for subjects 1–3. This accuracy was accomplished using as little as 133 seconds, and no more than eight minutes, of training data in each session. Furthermore, two of the subjects in this study (subject 1 and 3) had severe upper limb weakness contralateral to ECoG recording grids, arising from lesions of brain structures critical for motor control. Although subject 2 did not have contralateral limb weakness, he had a lesion of parietal lobe structures that participate in visually guided reaching and grasping movements of the upper limb [50]–[52]. Our findings indicate that the first PC of movement is robustly represented in the ipsilateral hemisphere, even in the face of damaged sensorimotor systems.

We found that high frequency signal features such as high gamma power tended to have a positive correlation with the first PC of movement, while low frequency features such as delta generally had a negative correlation. This result agrees with previous studies [10], [11], which have found that functional activation of cortex, including sensorimotor cortex, is accompanied by an event-related increase in power in the gamma band (>30 Hz) and an event-related decrease in power in lower frequencies, especially alpha (8–13 Hz) and beta (15–25 Hz) bands. The presence of this phenomenon in ipsilateral cortex agrees with results found in [43]. While typically not as robust as theta or mu, the delta band displayed notable power suppression during movements. The filters we employed were of an exceedingly high order (400), providing a roll-off steep enough to discount the possibility of bleed over from other low frequency bands. The LMP, however, did not consistently have a positive or negative correlation with the reaching movements.

There was substantial variability as to which spectral components of the ECoG signals were most predictive of the movement trajectory. For example, in subject 1 the mu band models achieved statistically higher decoding accuracies for the first PC of movement than did those constructed from high gamma 1 features; the opposite was true in subject 2, and both model types performed equivalently in subject 3. This variability in the performance of different ECoG spectral features across subjects suggests that an agnostic approach may be necessary when evaluating and selecting signal features for modeling ipsilateral movement kinematics, particularly in the face of different lesion locations and types. Much of the variability we observed was likely due to substantial differences in lesion size and location across our three subjects. Although patients with motor impairments do not frequently undergo intracranial monitoring, future studies might accumulate enough subjects to determine the spectral features that yield the best performance across subjects. Better yet, this might make it possible to better characterize the relationship between lesion characteristics (e.g. extracted from MRI via voxel based morphometry) and the performance of different spectral features. For example, more intact motor and premotor cortices in subjects 2 and 3 may have allowed for better decoding with high gamma power.

It is also possible that compensatory movements with the contralateral hemibody presented a confound [31], [34]. Indeed, reaching movements impart forces which are often accompanied by postural changes throughout the body, presenting a potential confound in any study of this sort. Our results should therefore be interpreted as the result of a movement involving complex dynamics in systems throughout the body.

The results depicted in Fig. 6 show that neural feature changes in subject 1 occurred following the onset of movement. This delayed change suggests that these neural responses arose chiefly from processing sensory feedback, perhaps because ipsilateral motor pathways were lesioned. Specifically, cortical lesions from prior resections in subject 1 probably involved hand and arm motor areas of the primary motor cortex. In contrast, subject 2 did not have motor deficits and the motor deficits in subject 3 were associated with a lesion in the left internal capsule. Unlike neural feature changes in subject 1, neural feature changes in subjects 2 and 3 preceded movement onset.

We found that decoding performance saturated with a small number of model inputs. That is, a few signal features at a few recording sites yielded the best performance, and the addition of more signal features and/or recording sites did not significantly improve performance. This finding could reflect a very coarse neural representation for ipsilateral limb movement that is widely distributed across sensorimotor systems typically specialized for contralateral limb movement. Alternatively, it could reflect transcallosal activation of these systems by homologous systems in the contralateral hemisphere. In this way, activity in sensorimotor cortex contralateral to movement (e.g. left hemisphere controlling right hand in subject 1 and 2) could modulate the activity of sensorimotor cortex ipsilateral to movement (e.g. in right hemisphere in subject 1 and 2). The encoding of this modulation during reaching movements might also be coarse, serving primarily to coordinate the movements of the two arms during reaching. Such explanations are necessarily highly speculative. The spatial resolution and anatomical distribution of our ECoG recordings were not sufficient to test these hypotheses. Nevertheless, our findings indicate that only a few electrodes are needed to achieve reach decoding from ECoG features, and that these electrodes qualitatively correspond to sensorimotor cortex as identified by ESM (Fig. 1).

We found that decoding of the second and third PCs of the movement kinematics was significantly worse than decoding of the first PC (*p*<0.05, ANOVA with Dunn-Sidak post-hoc), and that all but one of the models built for the second and third PCs were indistinguishable from chance. One possible explanation is that the second PC captured bi-directional movements in the lateral dimension, i.e. to the left or to the right (Fig. 4). The third PC qualitatively appeared to contain the most noise, and also often contained some bidirectional movements. These PCs stand in contrast to the first PC, in which movements only resulted in unidirectional deflections. One possible explanation for the poor accuracy with the second PC is that it is not the direction of movement, but instead its magnitude that is being decoded. It is possible that the correlation between movement *effort* and neural activity captured by ECoG is highly robust, such that it can even be captured in ipsilateral sensorimotor systems, even when these brain systems have been damaged. The directionality of movement, however, may present a greater challenge for decoding, especially under the circumstances of the subjects in this study. Whether the directionality of naturalistic reaching movements can be decoded from ECoG under more optimal conditions remains to be seen. Previous efforts [31], [44] have shown it is possible to decode hand movements from intact ipsilateral cortex in a constrained one- or two-dimensional experimental environment. However, decoding the directionality of movements may be more challenging in the setting of natural 3D movements, especially when performed by subjects with lesions of sensorimotor systems. While a preliminary analysis with ANN's did not improve decoding accuracy, there may be non-linearities within the data that could allow for directionality to be better decoded.

The ability to decode motor commands from ipsilateral sensorimotor systems, even when lesioned, may facilitate the development of brain-machine interfaces (BMIs) for a wider population of patients with upper limb paralysis from brain lesions such as stroke, trauma, or surgical resection of a tumor. For example, upper limb function could be partially restored by low dimensional control of a neuroprosthetic device in which ECoG electrodes are implanted over healthy ipsilateral cortex. Alternatively, electrodes could be implanted in and around cortical sensorimotor areas that have been damaged, in order to control the affected, contralateral upper limb. An important advantage of this approach is that it avoids the risks of surgically implanting chronic indwelling electrodes in healthy cortical areas controlling the patient's only functioning upper limb. In addition, implantation of ECoG electrodes in the lesioned hemisphere can be done in conjunction with other invasive interventions, such as infusions of stem cells and growth factors encouraging neurogenesis and neuroplasticity, thereby sharing the risk with other experimental therapies.

Our results suggest that it may also be possible to bootstrap a BMI system controlling contralateral arm movements by first using it to control ipsilateral arm movements. For example, the movements of the ipsilateral limb under BMI control could be mirrored in the contralateral arm using robotic assistance or functional electrical stimulation. This could provide an opportunity for development of contralateral arm control through functional reorganization and even neurogenesis. The neural activity generated while training the BMI could potentially facilitate neurobiological interventions through activity-dependent neuroplasticity, allowing the patient to eventually wean off ipsilateral arm control as control of the contralateral arm is restored. This might expedite the training process for BMIs by exploiting the more intact movements of the patient's unaffected limb.

## Supporting Information

S1 Table **Extrema of hand position relative to the shoulder in each dimension.** Units are in cm, e.g. S1's hand position moved between 21.6 cm below their shoulder and 15.2 cm above it.
(DOCX)

S2 Table **Experimental session details.**
(DOCX)

S1 Video **Actual (solid) and predicted (transparent) 3d hand position for subject 1.** Any time delays that would be induced by our offline methods have not been included. The sphere denotes the position of the target.
(MP4)

S2 Video **Actual (solid) and predicted (transparent) 3d hand position for subject 2.** Any time delays that would be induced by our offline methods have not been included.
(MP4)

S3 Video **Actual (solid) and predicted (transparent) 3d hand position for subject 3.** Any time delays that would be induced by our offline methods have not been included.
(MP4)
