# Supplementary material for: Coarse Electrocorticographic Decoding of Ipsilateral Reach in Patients with Brain Lesions
Source: PLoS One. 2014 Dec 29;9(12):e115236. doi: 10.1371/journal.pone.0115236 (PMC4278860; doi:10.1371/journal.pone.0115236)
Supplement: S1 Table — Extrema of hand position relative to the shoulder in each dimension. Units are in cm, e.g. S1's hand position moved between 21.6 cm below their shoulder and 15.2 cm above it. (DOCX) [file pone.0115236.s001.docx]

| S1, Session 1 | | | S1, Session 2 | | |  |  |  |
| --- | --- | --- | --- | --- | --- | --- | --- | --- |
| Height | Depth | Lateral | Height | Depth | Lateral |  |  |  |
| -21.6 | 43.7 | -8.4 | -22.4 | 44.9 | -12.3 |  |  |  |
| 15.2 | 64.1 | 27.0 | 15.9 | 63.7 | 24.9 |  |  |  |
|  |  |  |  |  |  |  |  |  |
| S2, Session 1 | | | S2, Session 2 | | | S2, Session 3 | | |
| Height | Depth | Lateral | Height | Depth | Lateral | Height | Depth | Lateral |
| -7.4 | 26.7 | -12.8 | -7.2 | 28.4 | -8.7 | -6.3 | 22.7 | -15.4 |
| 17.8 | 42.9 | 12.0 | 17.7 | 43.1 | 11.2 | 17.2 | 41.8 | 16.0 |
|  |  |  |  |  |  |  |  |  |
| S3, Session 1 | | | S3, Session 2 | | |  | | |
| Height | Depth | Lateral | Height | Depth | Lateral |  |  |  |
| -22.3 | 24.6 | -17.8 | -22.6 | 26.5 | -14.1 |  |  |  |
| 26.1 | 44.5 | 15.6 | 27.0 | 46.5 | 22.6 |  |  |  |
|  | | | | | | | | |

**Supplementary Table 1.** Extrema of hand position relative to the shoulder in each dimension. Units are in cm, e.g. S1’s hand position moved between 21.6 cm below their shoulder and 15.2 cm above it.
