# Supplementary material for: Coarse Electrocorticographic Decoding of Ipsilateral Reach in Patients with Brain Lesions
Source: PLoS One. 2014 Dec 29;9(12):e115236. doi: 10.1371/journal.pone.0115236 (PMC4278860; doi:10.1371/journal.pone.0115236)
Supplement: S2 Table — Experimental session details. (DOCX) [file pone.0115236.s002.docx]

| Subject, Session | Number  of Reaches | Session Length (s) | Minimum Intertrial Interval (s) | Maximum Intertrial Interval (s) |
| --- | --- | --- | --- | --- |
| S1, 1 | 22 | 164 | 1.5 | 5.1 |
| S1, 2 | 21 | 163 | 1.7 | 6.6 |
| S2, 1 | 31 | 206 | 1 | 9.3 |
| S2, 2 | 23 | 131 | 1.4 | 4.6 |
| S2, 3 | 30 | 213 | 1.4 | 11.5 |
| S3, 1 | 42 | 463 | 1.3 | 16 |
| S3, 2 | 32 | 280 | 1.5 | 9.25 |
|  | | | | |

**Supplementary Table 2.** Experimental session details.
